# Lymphomatoid papulosis type D with γδ phenotype evolving from pityriasis lichenoides in a pediatric patient

**DOI:** 10.1016/j.jdcr.2026.04.028

**Published:** 2026-04-23

**Authors:** Prameyuda Watchirakaeyoon, Panitta Sitthinamsuwan, Wanee Wisuthsarewong, Manasmon Chairatchaneeboon

**Affiliations:** a Department of Dermatology, Faculty of Medicine Siriraj Hospital, Mahidol University, Bangkok, Thailand; b Department of Pathology, Faculty of Medicine Siriraj Hospital, Mahidol University, Bangkok, Thailand; c Department of Pediatrics, Faculty of Medicine Siriraj Hospital, Mahidol University, Bangkok, Thailand

**Keywords:** lymphomatoid papulosis, LyP type D, γδ T-cell phenotype, PLC–PLEVA–LyP spectrum

## Introduction

Lymphomatoid papulosis (LyP) is a chronic, relapsing, self-remitting papulonodular skin disorder within the spectrum of primary cutaneous CD30-positive T-cell lymphoproliferative disorders (CD30^+^ lymphoproliferative disorder). Lesions are typically less than 2 cm, but larger or ulcerated papulonecrotic lesions may occasionally occur.^1^ Despite an excellent prognosis, up to 20% of cases are associated with secondary lymphoid neoplasms, necessitating long-term follow-up.^2^

LyP comprises histopathologic subtypes A to E, with an additional proposed subtype F, and a molecular subtype with DUSP22–IRF4 rearrangement.^2^^,^^3^ Subtype A is the most prevalent in children, while subtype D is rare.^4^ LyP type D shows epidermotropic infiltrates of pleomorphic, CD8^+^, CD4^−^ cytotoxic T lymphocytes and may clinically and histologically overlap with pityriasis lichenoides et varioliformis acuta (PLEVA) and other cutaneous lymphomas, posing diagnostic challenges.^5^

We report a 13-y-old boy with a 5-y history of recurrent erythematous papules initially diagnosed as pityriasis lichenoides chronica (PLC) and PLEVA. The disease was refractory to multiple therapies, and a subsequent biopsy established the diagnosis of LyP type D. Low-dose methotrexate (MTX) at a dose of 7.5 mg weekly resulted in rapid clinical improvement within 2 wk.

## Case report

A 13-year-old Thai boy presented with a 5-y history of recurrent, nonpruritic erythematous papules on the trunk and extremities. General physical examination was unremarkable. A skin biopsy from the back showed lichenoid interface dermatitis with focal parakeratosis and extravasated red blood cells, supportive of PLC. Initial treatment with oral erythromycin and topical corticosteroids was ineffective, and the lesions waxed and waned.

**Fig 1:**
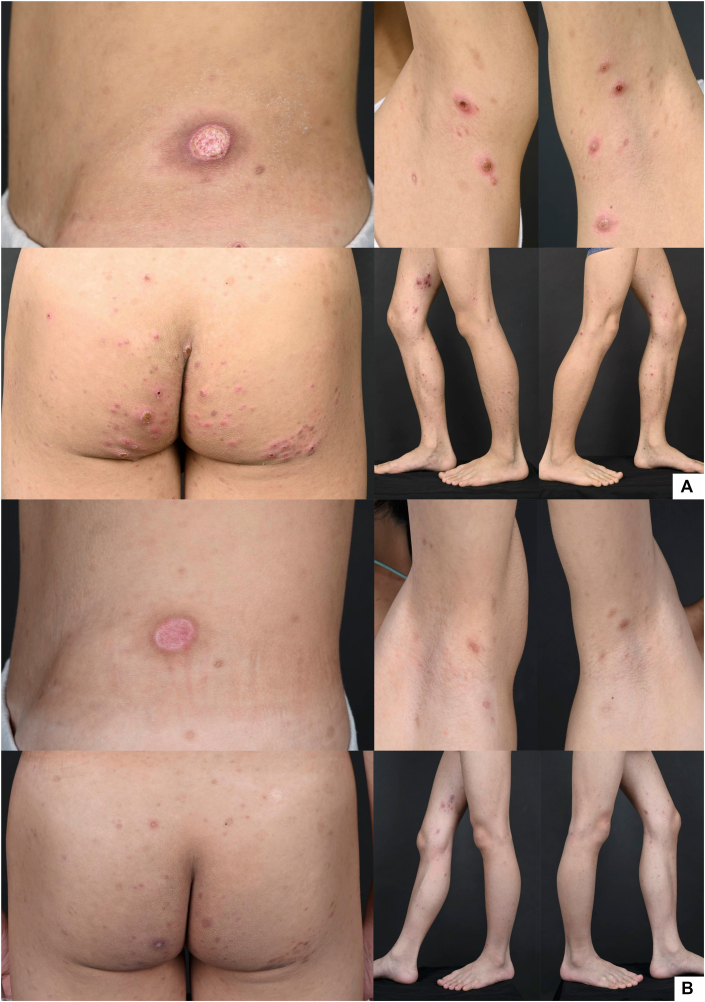
Clinical images of LyP type D with γδ phenotype showing multiple erythematous papules, some with central necrosis. **(A)** Before MTX therapy. **(B)** After MTX therapy. *LyP,* Lymphomatoid papulosis; *MTX,* methotrexate.

Four years after onset, the eruption progressed to widespread papules with ulcers and hemorrhagic crusts. A second biopsy from the left forearm revealed ulceration with scale crust, parakeratosis, and marked lichenoid interface dermatitis with extravasated red blood cells, compatible with PLEVA. Narrowband ultraviolet B phototherapy and doxycycline were initiated, followed by systemic corticosteroids, without clinical improvement (Fig 1, *A*). Methotrexate was deferred because of the patient’s age and risk - benefit considerations.

**Fig 2:**
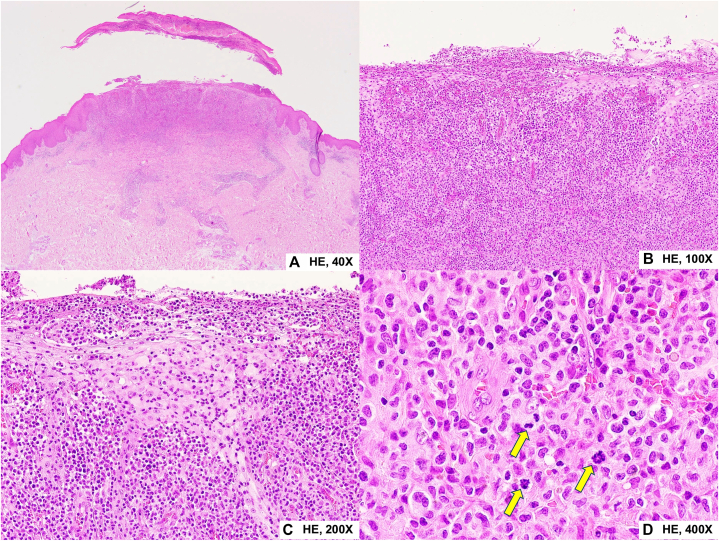
Histopathologic findings. **(A)** Epidermal erosion with scale crust formation (H&E, × 40). **(B)** Dense dermal lymphoid infiltrate (H&E, × 100). **(C)** Epidermotropism by atypical lymphoid cells (H&E, × 200). **(D)** Small-to medium-sized atypical mononuclear cells with irregular nuclear contours and atypical mitotic figures (*arrows*) (H&E, × 400).

**Fig 3:**
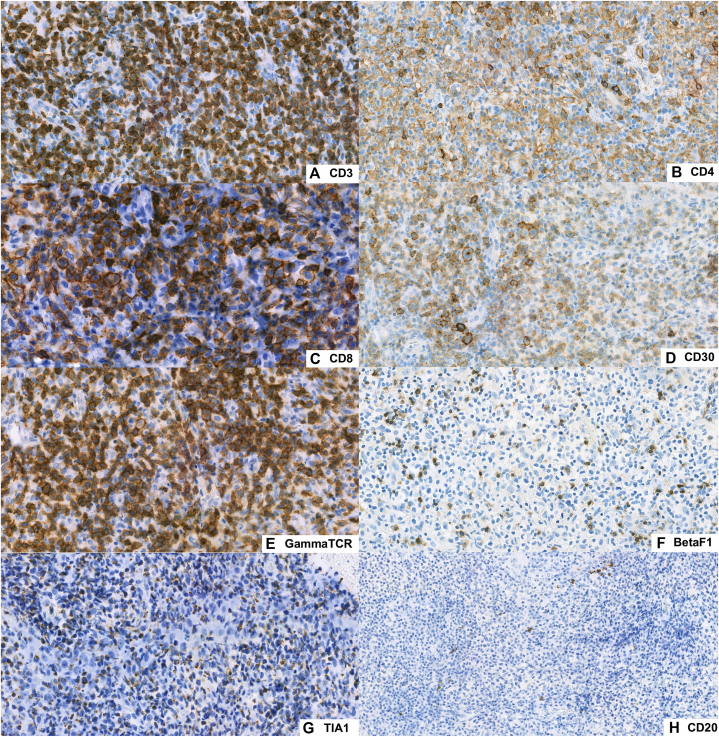
Immunohistochemistry. Tumor cells were positive for CD3 **(A)**, CD8 **(C)**, CD30 **(D)**, TIA-1 **(G)**, and gamma-TCR **(E)**, weakly positive for CD4 **(B)** and negative for beta-F1 **(F)** and CD20 **(H)**. *TCR,* T-cell receptor. *TIA-1*, T-cell intracellular antigen-1.

A third biopsy from an ulcerated papule on the right thigh revealed a nodular lymphoid infiltrate in the dermis with overlying surface erosion and necrosis. The infiltrate consisted predominantly of small-to medium-sized lymphoid cells with occasional large, atypical cells and scattered mitotic figures. Epidermotropism by small-to medium-sized lymphoid cells was noted, with a background inflammatory infiltrate consisting of histiocytes, neutrophils, and eosinophils (Fig 2). Immunostaining demonstrated that the atypical lymphoid cells were positive for CD3, CD8, CD30, TIA-1 and TCR-γ, weakly positive for CD4 and negative for CD20, beta-F1, CD56, CD1a and parvovirus B19. CD30 highlighted the medium-to large-sized atypical cells, comprising approximately 50% of tumor cells. Epstein-Barr virus-encoded RNA in situ hybridization was negative. The histopathologic and immunohistochemical findings supported the diagnosis of cutaneous CD30^+^ lymphoproliferative disorder with γδ phenotype, consistent with LyP type D (Fig 3).

Systemic evaluation, including complete blood count, liver and kidney function tests, lactate dehydrogenase level, serologic markers for hepatitis B and C, chest radiography, positron emission tomography–computed tomography, and bone marrow biopsy, revealed no evidence of extracutaneous involvement.

Molecular analysis demonstrated identical clonal T-cell receptor (TCR)-γ gene rearrangements, with negative TCR-β results, in both the earlier PLEVA lesion and the subsequent LyP type D lesion, confirming a shared, persistent clonal T-cell population.

Treatment with oral MTX (7.5 mg/wk) resulted in marked clinical improvement within 2 wk (Fig 1, *B*). Maintenance therapy at 5-7.5 mg/wk has provided sustained clinical improvement during continued follow-up.

## Discussion

LyP belongs to the spectrum of primary cutaneous CD30^+^ lymphoproliferative disorders and is frequently misdiagnosed as PLEVA because of overlapping clinicopathologic features.^2^^,^^4^^,^^6^ Increasing evidence suggests that PLC, PLEVA, and LyP represent a biologic spectrum rather than distinct entities, with clinical and histopathologic differences corresponding to disease severity at different time points.^5^

According to the World Health Organization–European Organization for Research and Treatment of Cancer classification incorporated into the WHO fifth edition (2022), LyP comprises 5 subtypes (A–E).^7^ Additional variants, including follicular LyP (proposed type F) and DUSP22–IRF4–rearranged LyP, have also been described.^2^ LyP type D, first recognized in 2010, predominantly affects adults and is rarely reported in children. Histologically, it is characterized by epidermotropic infiltrates of pleomorphic CD30^+^, CD8^+^, CD4^-^ cytotoxic T lymphocytes, as observed in our patient.^4^ A CD8^+^ cytotoxic phenotype is also commonly observed in PLEVA, supporting the concept that these entities may represent related processes. The identification of identical TCR-γ clones in our case further supports this possible biological continuum. Clonal TCR gene rearrangements have been reported in both LyP (22% to 100%)^2^ and PLEVA (55% to 65%).^8^ TCR-γ gene rearrangement assays have been used more frequently than TCR-β chain analysis because they are technically less complex and detect rearrangements in both αβ and γδ T-cell populations. Although most CD8^+^ cytotoxic T cells are derived from the αβ T-cell lineage, our LyP type D case demonstrated a γδ T-cell phenotype.

A γδ T-cell phenotype has been reported in a minority of LyP type D cases and may closely mimic primary cutaneous γδ T-cell lymphoma histologically. Badje et al demonstrated that, despite striking histopathologic similarity, γδ-positive LyP follows an indolent, LyP-like clinical course.^9^ Calcaterra et al also described pediatric cases with PLEVA-like features evolving into LyP type D.^5^ However, sequential PLC–PLEVA–LyP progression with a γδ phenotype, as observed in our patient, remains exceedingly rare.

The immunohistopathologic differential diagnoses include CD30-rich PLEVA, an inflammatory variant with numerous CD30-positive T cells, and primary cutaneous aggressive epidermotropic CD8-positive cytotoxic T-cell lymphoma.^3^^,^^4^ In our patient, a relapsing–remitting, self-healing course with medium-to large-sized CD30-positive lymphocytes argued against CD30-rich PLEVA,^8^ while the absence of systemic symptoms and an indolent clinical course argued against primary cutaneous aggressive epidermotropic CD8-positive cytotoxic T-cell lymphoma.^4^

Treatment of LyP is tailored by disease extent and severity. Observation or topical and intralesional corticosteroids may be sufficient for localized disease. Phototherapy and low-dose MTX is used for more widespread or recurrent disease.^2^^,^^6^ In our case, MTX at 5-7.5 mg/wk resulted in a positive response during long-term follow-up. In severe or refractory cases, systemic retinoids, interferon-α, and other immunomodulatory therapies have been reported, although supporting evidence remains limited. CD30-targeted therapy, particularly brentuximab vedotin, has shown promising results, whereas multiagent chemotherapy is generally discouraged.^2^^,^^10^ None of the available treatments have been shown to alter the natural disease course or to prevent recurrence or the development of associated malignancies.

Our case highlights the biologic continuum linking PLC, PLEVA, and LyP, demonstrated by identical TCR-γ clonality across sequential lesions. Recognition of this spectrum, particularly in pediatric patients with a γδ phenotype, is essential to avoid misdiagnosis and overtreatment and to guide appropriate long-term surveillance.

## Conflicts of interest

None disclosed.

## References

[1] Mark E., Kempf W., Guitart J. (2024). Lymphomatoid papulosis with T-cell receptor-gamma Delta expression: a clinicopathologic case-series of 26 patients of an underrecognized immunophenotypic variant of lymphomatoid papulosis. Am J Surg Pathol.

[2] Martinez-Cabriales S.A., Walsh S., Sade S., Shear N.H. (2020). Lymphomatoid papulosis: an update and review. J Eur Acad Dermatol Venereol.

[3] Willemze R., Cerroni L., Kempf W. (2019). The 2018 update of the WHO-EORTC classification for primary cutaneous lymphomas. Blood.

[4] Saggini A., Gulia A., Argenyi Z. (2010). A variant of lymphomatoid papulosis simulating primary cutaneous aggressive epidermotropic CD8+ cytotoxic T-cell lymphoma. Description of 9 cases. Am J Surg Pathol.

[5] Calcaterra V., Cavalli R., Croci G.A. (2022). Type D lymphomatoid papulosis with pityriasis lichenoides et varioliformis acuta-like features in a child with parvovirus infection: a controversial diagnosis in the spectrum of lymphoid proliferations: case report and literature review. Ital J Pediatr.

[6] Blanchard M., Morren M.A., Busschots A.M. (2024). Paediatric-onset lymphomatoid papulosis: results of a multicentre retrospective cohort study on behalf of the EORTC Cutaneous Lymphoma Tumours Group (CLTG). Br J Dermatol.

[7] Alaggio R., Amador C., Anagnostopoulos I. (2022). The 5th edition of the World Health Organization classification of haematolymphoid tumours: lymphoid neoplasms. Leukemia.

[8] Kempf W., Kazakov D.V., Palmedo G., Fraitag S., Schaerer L., Kutzner H. (2012). Pityriasis lichenoides et varioliformis acuta with numerous CD30(+) cells: a variant mimicking lymphomatoid papulosis and other cutaneous lymphomas. A clinicopathologic, immunohistochemical, and molecular biological study of 13 cases. Am J Surg Pathol.

[9] Badje E.D., Tejasvi T., Hristov A. (2019). γδ lymphomatoid papulosis type D: a histologic mimic of primary cutaneous γδ T-cell lymphoma. JAAD Case Rep.

[10] Kempf W., Pfaltz K., Vermeer M.H. (2011). EORTC, ISCL, and USCLC consensus recommendations for the treatment of primary cutaneous CD30-positive lymphoproliferative disorders: lymphomatoid papulosis and primary cutaneous anaplastic large-cell lymphoma. Blood.

